# Effect of sterile ice water versus menthol spray on thirst symptoms of fasted children in the intensive care unit: A prospective cohort study

**DOI:** 10.1097/MD.0000000000033315

**Published:** 2023-03-24

**Authors:** Fangyan Ma, Haiting He, Banghong Xu, Jing Zhou, Kai Pu

**Affiliations:** a Department of Cardiothoracic Surgery, Children’s Hospital of Nanjing Medical University, Nanjing, China; b Department of Nursing, Children’s Hospital of Nanjing Medical University, Nanjing, China.

**Keywords:** care, children, ice, ICU, menthol, nursing, spray, thirst

## Abstract

Thirst is a very common symptom in fasted children in intensive care unit (ICU). This study aimed to evaluate the effect of sterile ice water versus menthol spray in ICU fasted children, to provide insights to the clinical care of fasted children.

**Methods:**

The children admitted to the ICU of our hospital from June 1, 2021 to August 31, 2022 and needed to fast were included. Children were randomly assigned to the ice water group or menthol group. We evaluated and compared the thirst distress scale (TDS), oral mucosa wetness scale (OMWS), children medical fear scale (CMFS), numerical rating scale (NRS), unstimulated whole saliva (UWS) flow rate between 2 groups.

**Results:**

A total of 139 children were included, involving 69 children in ice water group and 70 children in menthol group. There were no significant differences in the baseline characteristics, TDS, OMWS, OMWS, CMFS, and NRS score, UWS flow rate before intervention between ice water group and menthol group (all *P* > .05). After intervention, the TDS, OMWS, NRS score of menthol group was statistically less than that of ice water group (all *P* < .05), the UWS flow rate of menthol group was statistically higher than that of ice water group (*P* = .034).

**Conclusions:**

Compared with ice water spray, menthol spray may be more beneficial to relieve the thirst and increase the comfort in ICU fasted children. Future studies with larger sample size and rigorous design are needed to evaluate the effects and safety of ice water and menthol spray in the nursing care of children.

## 1. Introduction

The intensive care unit (ICU), as the main place for hospitals to treat critically ill patients, not only provides emergency care conditions for patients, but also brings great pressure to patients. The noise of instruments, the large number of medical staff, and the continuous artificial lighting are all sources of stress for ICU patients, which often cause various discomfort during hospitalization and even after leaving the ICU.^[1–3]^ Some studies^[4,5]^ have pointed out that thirst is the strongest and most common type of discomfort in ICU, and it is one of the biggest sources of stress for ICU patients. This is mainly due to the fact that critically ill patients often lose too much body fluid due to surgical intervention and renal insufficiency, and the effective circulating blood volume is insufficient, so thirst is very easy to occur in ICU patients.^[6]^ Some studies^[7,8]^ have shown that the risk factors of thirst include gastrointestinal diseases, inability to drink water by mouth, and the use of high-dose diuretics and opioid analgesics. However, patients in ICU who fast for surgery, disease or treatment cannot meet the basic needs of drinking water, they will also feel thirsty, and their thirst-associated pain is more obvious.^[9]^

ICU patients often need to fast due to therapeutic factors. The incidence of thirst symptoms in ICU patients is high, which brings serious problems to ICU patients. Currently medical staff have gradually improved the recognition and intervention of thirst in ICU patients. However, at present, there are few studies on thirst in ICU fasted children. As a special population in ICU, fasted children are easy to be ignored in clinical work because their age, experience and stress state are different from adults.^[10,11]^ Particularly, the separation anxiety, fear of medical treatment, pain and other symptoms of fasting children are more obvious, the treatment compliance and subjective comfort are reduced,^[12,13]^ thus may affect the recovery of the disease and the hospital stay, which should attract the attention of medical care providers. Therefore, we aimed to evaluate the effect of sterile ice water versus menthol spray on thirst symptoms of fasted children in ICU, to provide reliable evidence for reducing the thirst and improving the comfort and prognosis of fasted children in ICU.

## 2. Methods

This study was a prospective cohort design. The study protocol had been analyzed and approved by the ethical committee of our hospital with approval number: 202207155-1. And written informed consents had been obtained from all the guardians of included children.

### 2.1. Sample size calculation

The sample size was calculated by the sample size estimation formula of 2 groups of independent samples.^[14]^ The formula was as follows: $n_{1}=n_{2}=\frac{2{\left(u_{\alpha }+u_{\beta (1)}\right)}^{2}\sigma^{2}}{\delta^{2}}$. In this formula, n was the required content of each group of samples; σ^2^ was the population standard deviation (assuming that the standard deviation of 2 populations was equal), which could be expressed by the mean deviation of 2 samples; δ^2^ was the difference between the means of the 2 groups. *α* and *β* was the first type error rate and the second type error rate. We assumed that *α* = 0.05, *β* = 0.20, The minimum sample size for each group was 56 cases. Considering the rigor of the study, the convenience of clinical data collection, and the 20% sample loss rate, the final sample size should be 136 cases with 68 cases in each group.

### 2.2. Study population

We selected the children who were hospitalized in the ICU of our hospital from June 1, 2021 to August 31, 2022 and needed to fast as the study population. The inclusion criteria of children in this study were as following: children who needed to fast for treatment according to the doctor advice; children aged 8 to 14 years; children who were conscious with Ramsay sedation scores score ranging from −1 to +1; the length of ICU stay was more than 24 hours; and the children and guardians were well informed and agreed to participate in this study. The exclusion criteria of children in this study were as following: children with obvious oral infection associated with salivary pancreatitis, chemotherapy, or Sjogren syndrome, which might significantly influence the thirst feelings of children; those who have consciousness barriers and were unwilling to communicate; children whom were unwilling to participate in this study.

### 2.3. Equipment

Spray bottle: The capacity was 20 mL, and the amount of spray liquid was 0.5 mL per pressing. Manufacturer: Miniso (Nanjing Co., Ltd.); Sterilized water: The capacity was 500 mL and placed in the refrigerator at 4 °C. And it was be directly loaded into the selected watering to make ice water spray. Manufacturer: Shiyao Yinhu Pharmaceutical Co., Ltd. Menthol: The menthol for food flavor was soluble in water to prepare the menthol spray liquid. The preparation method was as following: We took l mL of menthol, added it to 500 mL sterilized water at 4 °C in cold storage, shake well enough to prepare a saturated solution of menthol with a concentration of 2‰. Menthol manufacturer: Nanjing Hua Yang Essence and Fragrance Industry Co., Ltd.

### 2.4. Interventions

The included children were randomly assigned to the ice water group and menthol group accordingly. Ice water group: confirming the children were conscious, we began to intervene for 3 timepoints, namely 0 minute, 15 minutes, and 30 minutes after starting spray. Before operation, we sprayed the air for several times to remove the air from the spout, so as to make the spray even. Then we asked the child to open his mouth and lift his tongue, sprayed it to the sublingual area, the mucosa in the left and right cheeks, and the tongue surface, and asked the child to close his mouth for 5 minutes for every intervention. After 40 minutes, the pain degree of thirst, medical fear and comfort degree of the children were scored respectively.

Menthol group: The evaluation and intervention period was the same as that of Ice water group. The prepared ice menthol spray was used to spray the oral cavity. The spraying method was the same as that of Ice water group, and the children were instructed to close their mouths for 5 minutes. After 40 minutes, the pain degree of thirst, medical fear and comfort degree of the children were scored respectively.

### 2.5. Outcome assessments

#### 2.5.1. Thirst Distress Scale (TDS).

TDS^[15]^ consists of 3 dimensions and 6 items, including duration of thirst (2 items), frequency of thirst (2 items) and intensity of thirst (2 items). The Likert 4 subscale method is used in this scale. One point means irrelevant, and 4 points means very relevant. Children can choose the number that can represent their subjective feelings, and the total score is the evaluation result. The total score is 6~24 points, which respectively indicates that there is no discomfort to continuous thirst.

#### 2.5.2. Oral mucosa wetness scale (OMWS).

This scale^[16]^ evaluate the degree of wetness of oral mucosa according to the wetness of oral mucosa. The lips begin to crack and peel, the mouth is completely dry for 4 points, the lips and mouth are completely dry for 3 points, the mouth is wet and the lips is dry for 2 points, and the mouth and lips are wet for 1 point. The lower the score, the more moister the mouth.

#### 2.5.3. Children medical fear scale (CMFS).

CMFS^[17]^ consists of 4 dimensions and 14 items, including fear of medical environment (3 items), fear of medical operation (4 items), fear of interpersonal relationship (4 items) and fear of self (3 items). The Likert 3 subscale method is used in this scale. One point indicates no phobia, 2 points indicates some phobia, and 3 points indicates very phobia. Children can choose the number that can represent their subjective feelings, and the total score is the evaluation result. The total score is 14~42, which respectively indicates no fear to continuous fear.

#### 2.5.4. Numerical rating scale (NRS).

NRS^[18]^ was rated with a ruler printed with a total of 11 numbers from 0 to 10. 0 represents “no thirst,” 1 to 3 represents mild thirst, 4 to 7 represents moderate thirst, and 8 to 10 represents severe thirst. During the evaluation, the nurse explained the meaning of the ruler number to the patient in a unified language, so that the patient could tick the number representing the severity of his thirst. It has been reported that the internal consistency of thirst intensity of patients in ICU and anesthesia recovery room is good (Cronbach *α* = 0.82).

#### 2.5.5. Unstimulated whole saliva (UWS).

In this study, the cotton swab method was used to measure UWS flow rate.^[19]^ After the spray intervention, the saliva in the mouth was sucked dry with a cotton ball, and then 3 weighed dry cotton swabs were selected and placed under the tongue and parotid glands on both sides of the children respectively. The patient was instructed not to swallow, and the time was 2 minutes. When the cotton swab was taken out, the saliva on the tongue surface was sucked dry together, and then the gross weight was weighed, and then the value obtained was divided by 2, which was the value of static total saliva flow rate (mg/min). The UWS flow rate before and after intervention was compared between the 2 groups.

### 2.6. Statistical methods

In this study, SPSS 23.0 statistical software (IBM Company, Armonk, NY) was used for data statistical analysis. Descriptive statistics were used for general data of patients, frequency (%) was used for qualitative data, Chi square test or Fisher exact test was used for inter group comparison; mean ± standard deviation was used for quantitative data conforming to normal distribution, 2 independent samples *t* test was used for inter group comparison, and paired samples *t* test was used for intra group comparison. *P* < .05 meant that the difference was statistically significant between 2 groups.

## 3. Results

### 3.1. The characteristics of included children

**Figure 1.**
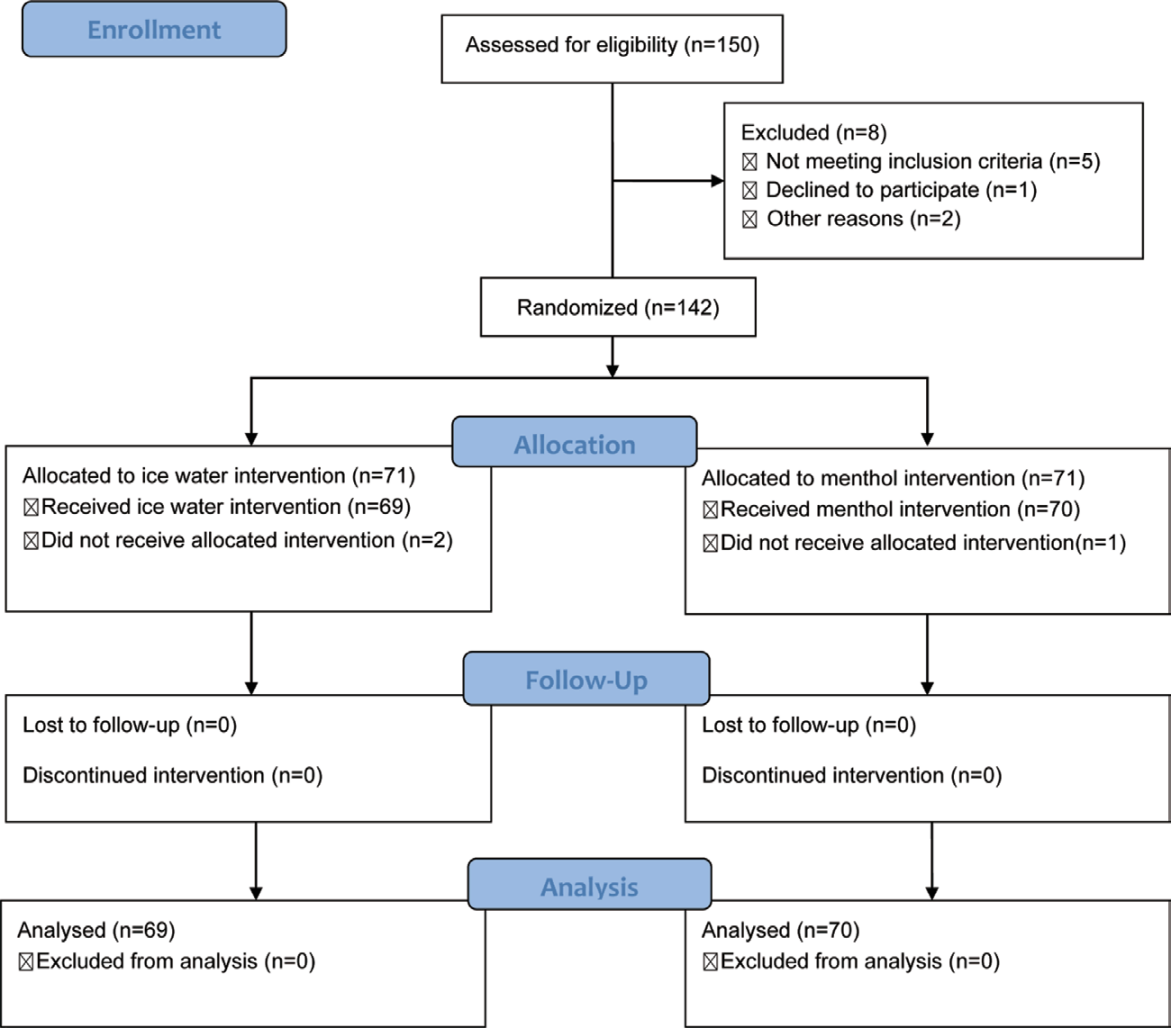
The CONSORT flow diagram of children inclusion.

A total of 150 fasted children were initially identified, 8 children were excluded because of not meeting the inclusion criteria, 3 children did not receive the allocated interventions. Finally, 139 children were included, with 69 children in ice water group and 70 children in menthol group (Fig. 1).

The characteristics of included children are presented in Table 1. There were no significant differences in the age, gender, body mass index, acute physiology and chronic health evaluation score, disease types, diuretics use, opioid use, hyponatremia, hypernatremia, blood ionized calcium level, blood sugar, antihypertensive therapy, duration of fasting and length of ICU stay between ice water group and menthol group (all *P* > .05).

**Table 1:** The characteristics of included ICU children.

Characteristics	Ice water group (n = 69)	Menthol group (n = 70)	*t*/*χ*^2^	*P*
Age (yr)	11.2 ± 3.2	11.1 ± 2.9	2.127	.104
Female/male	33/36	31/39	1.503	.087
BMI (kg/m^2^)	19.6 ± 3.1	19.6 ± 3.0	2.174	.116
APACHE-II score	18.7 ± 2.7	18.9 ± 3.1	3.005	.102
Disease types			2.482	.058
Cardiovascular disease	41 (59.42%)	44 (62.86%)		
Respiratory diseases	12 (17.39%)	13 (18.57%)		
Surgical disease	16 (23.19%)	13 (18.57%)		
Undergoing continuous renal replacement therapy	8 (11.59%)	8 (11.43%)	1.353	.117
Diuretic use	21 (30.43%)	20 (28.57%)	1.154	.081
Opioid use	14 (20.29%)	17 (24.29%)	1.612	.079
Hyponatraemia	3 (4.35%)	3 (4.29%)	1.009	.088
Hypernatraemia	5 (7.25%)	4 (5.71%)	1.281	.059
Blood ionized calcium level (mmol/L)	2.5 ± 0.7	2.4 ± 0.6	1.446	.274
Blood sugar level (mmol/L)	5.6 ± 2.4	5.8 ± 3.0	1.208	.116
Antihypertensive therapy	10 (14.49%)	9 (12.86%)	1.952	.083
Duration of fasting (h)	14.2 ± 4.5	14.2 ± 5.1	3.199	.121
Length of ICU stay (d)	5.5 ± 2.2	5.6 ± 2.8	2.018	.095

APACHE-II = acute physiology and chronic health evaluation, BMI = body mass index.

### 3.2. TDS score

As shown in Table 2, there was no significant difference in the TDS score before intervention between ice water group and menthol group (*P* = .095). After intervention, the TDS score of menthol group was statistically less than that of ice water group (*P* = .016), indicating that menthol spray may be more beneficial to reduce the thirst distress.

**Table 2:** The comparison of thirst distress scale scores.

	Ice water group (n = 69)	Menthol group (n = 70)	*t*	*P*
Before intervention	18.17 ± 3.53	18.12 ± 4.07	3.114	.095
After intervention	16.03 ± 2.15	14.09 ± 3.12	2.506	.016
*t*	2.105	2.531		
*P*	.043	.008		

### 3.3. OMWS score

As shown in Table 3, there was no significant difference in the OMWS score before intervention between ice water group and menthol group (*P* = .093). After intervention, the OMWS score of menthol group was statistically less than that of ice water group (*P* = .009), indicating that menthol spray may be more beneficial to increase the oral mucosa wetness.

**Table 3:** The comparison of oral mucosa wetness scores.

	Ice water group (n = 69)	Menthol group (n = 70)	*t*	*P*
Before intervention	3.44 ± 1.91	3.38 ± 1.02	1.452	.093
After intervention	2.43 ± 1.04	1.27 ± 1.12	1.028	.009
*t*	1.201	1.945		
*P*	.062	.022		

### 3.4. CMFS score

As shown in Table 4, there were no significant differences in the CMFS score before and after intervention between ice water group and menthol group (all *P* > .05).

**Table 4:** The comparison of children medical fear scale scores.

	Ice water group (n = 69)	Menthol group (n = 70)	*t*	*P*
Before intervention	31.05 ± 7.22	30.25 ± 6.79	6.244	.101
After intervention	28.53 ± 6.95	27.94 ± 7.03	5.916	.089
*t*	5.421	6.014		
*P*	.056	.095		

### 3.5. NRS score

As shown in Table 5, there was no significant difference in the NRS score before intervention between ice water group and menthol group (*P* = .112). After intervention, the NRS score of menthol group was statistically less than that of ice water group (*P* = .044), indicating that menthol spray may be more beneficial to reduce the thirsty feelings.

**Table 5:** The comparison of numerical rating scale cores.

	Ice water group (n = 69)	Menthol group (n = 70)	*t*	*P*
Before intervention	8.17 ± 3.53	8.23 ± 3.74	2.016	.112
After intervention	5.46 ± 2.01	3.14 ± 1.95	1.933	.044
*t*	1.255	1.402		
*P*	.049	.017		

### 3.6. UWS flow rate

As shown in Table 6, there was no significant difference in the UWS flow rate before intervention between ice water group and menthol group (*P* = .181). After intervention, the UWS flow rate of menthol group was statistically higher than that of ice water group (*P* = .034), indicating that menthol spray may be more beneficial to increase the UWS flow rate.

**Table 6:** The comparison of unstimulated whole saliva flow rate.

	Ice water group (n = 69)	Menthol group (n = 70)	*t*	*P*
Before intervention	0.26 ± 0.11	0.26 ± 0.14	1.094	.181
After intervention	0.49 ± 0.14	0.72 ± 0.22	1.217	.034
*t*	1.103	1.295		
*P*	.077	.041		

## 4. Discussion

Thirst is a subjective feeling that can trigger the desire of the human body to drink water, a manifestation of body imbalance, and one of the important factors influencing the patient comfort.^[20,21]^ The normal value of serum sodium concentration is 135 to 145 mmol/L. When the serum sodium concentration exceeds the normal range, hypernatremia will occur, and the pituitary osmoreceptor cells will dehydrate. The plasma alternating osmotic pressure is affected accordingly. As long as it is 1% to 2% higher than the normal range, it will stimulate the increase of excitatory output, thus triggering the release of antidiuretic hormone and the generation of thirst.^[22,23]^ When the balance of volume inside and outside cells is broken, the body will trigger a negative feedback system to trigger thirst signals to restore this balance.^[24]^ The pain of thirst in ICU fasting patients can cause anxiety, irritability, low treatment compliance, sleep disorders and other states.^[25]^ Some studies^[26,27]^ show that strong thirst lasting more than 24 hours will increase the risk of delirium. The experience of thirst discomfort will make ICU patients in a state of stress, significantly increase the patient oxygen consumption, increase the burden of organ metabolism, and affect the prognosis of patients.^[28]^ The painful experience of thirst will continue until the patient leaves the ICU, which will lead to a decrease in the patient comfort, an increase in dissociative anxiety, and an increase in fear, affecting the recovery of the disease.^[29,30]^ For children in ICU, prolonged fasting and water deprivation will significantly increase their psychological pressure, keep their bodies in an emergency state, delay their recovery, and cannot meet their comfort requirements.^[31]^ The results of this study have found that compared with ice water spray, menthol spray may be more beneficial to reduce the thirst distress and feelings, increase the oral mucosa wetness and UWS flow rate, menthol spray may be promoted in the clinical nursing care of fasted children in ICU.

Some studies^[32,33]^ have pointed out that, thirst can be relieved by “pre absorption” and “post absorption.” Before water is absorbed by the body, the liquid in the mouth and in the process of swallowing will stimulate the receptors in the mouth and pharynx, so that excitement will be transmitted to the brain to produce a sense of satisfaction for drinking water.^[34]^ After water is absorbed by the body, the thirst is relieved mainly by maintaining the balance of plasma osmotic pressure and blood volume.^[35]^ Previous studies^[36,37]^ have showed that through “pre absorption” stimulating the receptors of oropharynx to relieve thirst of such patients is a very effective intervention strategy. Compared with normal temperature stimulation, stimulation of oropharyngeal receptors with low temperature has a significant effect on relieving the symptoms of thirst in patients.^[38]^ It has been reported that cold stimulation can activate the TRPM8 cold receptor distributed in the oral mucosa, therefore leading to cold sensation.^[39]^ There will be more comfort without taking a lot of water, and the discomfort of thirst will also be alleviated.^[40]^

Many previous studies^[7,8]^ have shown that the local intervention of ice water spray is more effective than the cotton swab lip wetting method. Ice water spray will change the liquid into water mist, and under the action of the nozzle, it will quickly and evenly reach each part of the oral cavity. At the same time, the water mist can form a protective film to protect the oral mucosa, maximize the moist effect of the oral mucosa, reduce the occurrence of dry lips and whitening, thus reducing the occurrence of dry mouth and thirst.^[41]^ The spray range of ice water spray is up to about 40 cm, so as to ensure that the spray can reach the deep throat of the patient, produce a moist effect on the throat, and do not stimulate the deep throat of the patient, enhancing patient comfort.^[42]^ The decrease of saliva secretion is also the main reason for patients to feel thirsty. Ice water spray can act on the throat of patients to stimulate them to produce more saliva.^[43]^ Besides, it can also inhibit the release of antidiuretic hormone, reduce the frequency of patients’ thirst, thus reducing the number of additional interventions.^[44]^ The results of this study show that menthol spray may be more beneficial because it can stimulate the taste buds to produce a sense of coolness, and reduce the patient thirst and discomfort caused by the taste. The nourishing effect of liquid on the mouth and pharynx will also make thirsty patients feel happy and rewarded. There are osmoreceptors in the oral and throat mucosa. When patients feel the liquid in the mouth and throat, they can quickly relieve thirst. Besides, both cold and menthol stimulation can activate the transient receptor potential channel of oropharynx, so that stimulation can be transmitted to the cerebral cortex of children, resulting in reflex inhibition of thirst and pleasure satisfaction.^[45,46]^

This study has some limitations that are worth considering. First of all, this study is a single center study, involving a small number of children. Secondly, limited by the research conditions, the effect of this study on ice water and menthol is mainly judged by various scales, and there is still a lack of objective indicators to analyze the treatment effect. Thirst is primarily caused by an increase in fluid osmolality and decrease in blood volume. We did not regularly monitor the plasma osmolality and blood volume unless the children had obvious related symptoms, thus we lacked the data on the plasma osmolality and blood volume. Thirdly, we currently did not find any side effect associated with menthol spray, more long-term studies are needed to further evaluate the safety of menthol spray. Finally, this study did not apply blind methods to patients, nurses and outcome evaluators. It is necessary to conduct more high-quality researches with larger sample in the future to further analyze the effect and safety of ice water and menthol on fasting children.

## 5. Conclusions

In conclusion, we have found that compared with ice water spray, the menthol spray may be more advantageous to reduce the thirst distress and feelings, increase the oral saliva secretion and mucosa wetness. Menthol spray is a safe, convenient and effective method for preventing thirst, which may be worthy of promoted in clinical nursing practice for fasted children in ICU.

## Author contributions

**Data curation:** Kai Pu.

**Formal analysis:** Kai Pu.

**Funding acquisition:** Kai Pu.

**Investigation:** Fangyan Ma, Haiting He, Banghong Xu, Jing Zhou, Kai Pu.

**Methodology:** Kai Pu.

**Visualization:** Kai Pu.

**Writing – original draft:** Kai Pu.
